# Trust in Centralized Large-Scale Data Repository: A Qualitative Analysis

**DOI:** 10.1177/1556264619888365

**Published:** 2019-11-18

**Authors:** Reinder Broekstra, Judith Aris-Meijer, Els Maeckelberghe, Ronald Stolk, Sabine Otten

**Affiliations:** 1 University Medical Center Groningen, the Netherlands; 2 University of Groningen, the Netherlands

**Keywords:** trust, big data, biorepositories/biobanks, cohort study, decision making, qualitative methods, the Netherlands, justice/participant selection/inclusion/recruitment

## Abstract

Exponential increases in digital data and calls for participation in human research raise questions about when and why individuals voluntarily provide personal data. We conducted 36 in-depth interviews with ex-participants, participants, and nonparticipants in a biobank to identify key factors influencing trust in centralized large-scale data repository for human research. Our findings indicated that trust depends strongly on whether such data repository benefits the public, the interests of data collectors, the characteristics of the collected data, and application of informed consent for retaining control over personal data. Concerns about the aims and range of data repository appeared to influence withdrawal of participation. Our findings underscore ethical and practical issues relating to data collection and consent procedures in human research.

In recent years, there has been an exponential increase in digital data and calls for participation in human research through the provision of personal information for centralized large-scale data repositories, especially in the medical sciences. This phenomenon warrants an investigation of when and why individuals voluntarily provide data or withdraw their participation (Broekstra et al., 2017). The overall increase in digital data repository has raised concerns among experts as well as participants in centralized large-scale data repository about autonomy, privacy, and responsibility (Mittelstadt et al., 2016; Mittelstadt & Floridi, 2016; Sorani et al., 2015; Stricker, 2017). Innovative methods applied in centralized large-scale data repository and the linking of personal data, such as blood samples, to other types of health and nonhealth information permit an even wider range of data use than do traditional research methods (Prainsack & Buyx, 2013; Steinsbekk, Ursin, et al., 2013).

Such data usage, however, deviates from the ambit of current standards for safeguarding participants and nonparticipants against violations of integrity, such as standard informed consent and anonymization procedures, and methods of protecting data (Barocas & Nissenbaum, 2014; boyd & Crawford, 2012; Keymolen, 2016; Mittelstadt & Floridi, 2016; Robinson et al., 2013). Moreover, it raises questions regarding the confidentiality of personal data, possible stigmatization, and uncertainty about who has access to the data (boyd & Crawford, 2012; Gibson et al., 2017; Graeff & Harmon, 2002; Mittelstadt & Floridi, 2016; Rivera et al., 2017). Health and medical care will be prioritized in relation to science and innovation over the next 15 years (Eurobarometer, 2014). Therefore, a study of the different perspectives of stakeholders in human research entailing centralized large-scale data repositories on data sharing and trust is pertinent. Specifically, we compared the perspectives of ex-participants, participants, and nonparticipants in biobanks for facilitating human research in the medical sciences.

With the establishment of biobanks, concern has shifted from questions of privacy to those of trust (Kelley et al., 2015; Mittelstadt & Floridi, 2016). Biobanks store large quantities of biological specimens as well as data extracted from questionnaires and measurements. They are aimed at facilitating studies on patients with specific diseases or prospective studies on the onset and progress of chronic diseases (Krokstad et al., 2013; Scholtens et al., 2015; Sudlow et al., 2015; UK Biobank Coordinating Centre, 2007; van Staa et al., 2016). Studies of biobanks, entailing extensive data repository over a long duration, differ from small-scale or cohort studies. Centralized information storage in biobanks inevitably poses a threat to participants’ privacy. The decision to participate in a biobank entails accepting unknown short-term and long-term privacy risks concerning the confidentiality of personal data, the use of such data for unintended research purposes, or possible stigmatization (boyd & Crawford, 2012; Gibson et al., 2017; Graeff & Harmon, 2002; Mittelstadt & Floridi, 2016; Nobile et al., 2016; Rivera et al., 2017). This situation results in a social dilemma in which the collective interest of promoting the public good and the individual interest of keeping data or information private are opposed. Trust may reduce the complexity of this dilemma through general assumptions whereby an unfamiliar world or future is simplified, thus enabling decision making on risk taking (Luhmann, 1979). Studies have shown that trust in biobanks, health care providers, and other public institutions is a key factor in determining individuals’ willingness to participate in epidemiological research and to provide personal data to biobanks (Critchley et al., 2012; Gaskell et al., 2013; Hansson, 2005; Kaufman et al., 2009; Kettis-Lindblad et al., 2006; Lemke et al., 2010; Nobile et al., 2016; Rahm et al., 2013).

Currently, biobanks and other facilities used in human research attempt to enhance or change the complexity, volume, and nature of their data repositories to optimize them (Mittelstadt & Floridi, 2016). Researchers and data collectors seek to link individuals’ data to other types of personal data available within analyses of large data repositories to illuminate complex patterns and build predictive models (Barocas & Nissenbaum, 2014; Nuffield Council on Bioethics, 2015). These “big health data” can emerge out of detailed data revealing individuals’ characteristics, behaviors, and preferences, for example, through combined analyses of questionnaires and genomic, register, sensory, satellite, and social media data. Accordingly, voluntary participation in research in a context of openly shared information requires a greater degree of trust in the data repositories and their use in human research, compared with a context in which enrichment or sharing of data does not occur (Gibson et al., 2017; van Staa et al., 2016).

Trust, however, is a concept that is complex and difficult to grasp (Parks et al., 2015). Although trust seems to be a requirement for participation in research, its function within decision making regarding participation or withdrawal remains unclear. Moreover, the distinction between trust and distrust is still a matter of debate, with some scholars viewing trust and distrust as two ends of a single continuum, whereas others view them as distinct (Keymolen, 2016; Luhmann, 1979; Saunders et al., 2014). Trust can be defined as an individual’s willingness to be vulnerable without evaluating the behavior and intention of another person that automatically leads to a decision on whether or not to accept the risk (Ferrin et al., 2006; Mayer et al., 1995). Other scholars regard trust as a cognitive construct relating to the outcome of an individual’s reasoning process regarding their own vulnerability when evaluating others’ behaviors and intentions (Bhattacharya et al., 1998; Lewicki et al., 1998). The contradictory findings of studies on trust and biobanking lend support to both views. According to the findings of a study conducted in the United States in 2009 (*n* = 4,659 respondents), regardless of trust, privacy remains an important issue for potential participants in biobank studies, indicating the prevalence of automatic judgments (Kaufman et al., 2009). However, the findings of another study indicating that privacy concerns were less salient in decisions to participate in a biobank study when high levels of generalized trust existed support a conception of trust as a cognitive rational construct (Gaskell et al., 2013).

It is therefore important to understand why and under what conditions individuals are willing to trust a biobank with their personal data and to identify specific conditions that contribute to a reduction of this trust (Nobile et al., 2016; Petersen et al., 2014). In particular, ex-participants’ reasons for withdrawal can yield valuable insights into the process of trust building. A comparative study encompassing ex-participants, participants, and nonparticipants in a biobank that has introduced new methods for optimizing data repositories in an open context of information sharing would advance knowledge regarding the pathways that lead to the acquisition or loss of trust. Several studies have focused individually on ex-participants, participants, and nonparticipants (Critchley et al., 2016; Gaskell et al., 2013; Nobile et al., 2013; Steinsbekk, Ursin, et al., 2013), although empirical investigations of ex-participants are somewhat limited (Ridgeway et al., 2013). However, few studies have examined these groups simultaneously. Therefore, we explored the conditions under which individuals voluntarily entrust a biobank with their personal data and the reasons why trust sometimes decreases over time.

## Method

### Data Collection

We conducted semi-structured individual interviews, as they enable an in-depth exploration of respondents’ perceptions, events, and experiences relating to centralized large-scale data repository and research (non)participation. Moreover, their opinions and statements can be triangulated and clarified (Kvale, 2007, p. 11). We developed an interview topic guide and applied a narrative approach that left room for discussion of unanticipated themes. This narrative approach was partly derived from the method used in the DIPEx project (Ziebland & McPherson, 2006) that focused on personal experiences of health and illness.

The interview topic guide, shown in Table 1, was developed on the basis of six current themes identified in the scientific literature on (non)participation in biobanking, public goods, trust, and data sharing (Balliet & Van Lange, 2013; D’Abramo, 2015; Gaskell et al., 2013; Kaufman et al., 2009; Mittelstadt et al., 2016; Mittelstadt & Floridi, 2016; Nobile et al., 2016, 2013; Parks et al., 2013; Sorani et al., 2015; Steinsbekk, Myskja, & Solberg, 2013). The following themes were identified: (a) becoming a (non)participant, (b) objective aspects of participation (e.g., general feelings, tasks, and feedback), (c) subjective aspects of participation (e.g., expectations of accomplishment and feelings of identification), (d) understandings of and attitudes toward centralized data repository and linkage, (e) perceived benefits and threats relating to centralized data repository and linkage, and (f) decisions on whether or not to provide personal data. The interview topics were subsequently derived from these themes.

**Table 1.** Interview Topics and Illustrative Questions.

Topic	Example questions
Association with biobank	How did you get involved?
Decision of (non)participation	What were your considerations?
Reality	Was your experience as expected or not?
Future	Will you participate in Lifelines or in another centralized large-scale research project in the future?
Overall perception	How is participation in Lifelines perceived in general?
Tasks	How are the tasks related to participation in Lifelines perceived?
Feedback	What do you think of the feedback on your data you (would) receive?
Outcome hoped for	What motivates you to participate?
Benefits	What are the benefits and opportunities relating to participation?
Disadvantages	What are the risks and disadvantages relating to participation?
Definition	What are centralized large-scale data repositories?
Attitudes toward large-scale centralized data repositories	Are your attitudes toward collection, linking, and use of these data generally positive or negative?
Benefits of large-scale centralized data repositories	What are the benefits of collecting, linking, and using data?
Risks posed by large-scale centralized data repository	What risks are posed by the collection, linking, and use of data?
Personal balance of benefits and threats	From your personal perspective, how are these considerations balanced?
Societal balance of benefits and threats	From a societal perspective, how are these considerations balanced?

All of the interviews were conducted by one member of the research team with training and experience in interviewing techniques (R.B.). Current participants in Lifelines, a Dutch population-based biobank, were interviewed following their regular visits to the Lifelines research site. Most of the interviews were held at this site or in a room allocated for the interviews at the University Medical Center Groningen. All four ex-participants from Lifelines and eight of the fifteen nonparticipants were interviewed at their homes at their request. Each interview covered all of the topics in the guide and lasted between 30 and 65 min, depending on when saturation was reached in interviews. The interviews were audio-recorded with the consent of the interviewees. Only one interviewee denied permission for audio recording the interview, but allowed notes to be taken. All of the recordings were transcribed by an independent professional organization. The complete study design protocol received ethical approval from the Medical Ethics Review Board.

### Recruitment and Sampling

Maximum variation sampling was applied to recruit interviewees (Coyne, 1997). Consequently, a heterogeneous sample was obtained (Baker & Edwards, 2012; Mason, 2010). Between August and September 2016, a total of 36 individuals were interviewed: 17 had participated in Lifelines, 4 were ex-participants, and 15 did not participate in Lifelines. In this article, we refer to these distinct groups of interviewees as “participants,” “ex-participants,” and “nonparticipants.” Table 2 shows the characteristics of each of the interviewees. The average age of the interviewees was 45 years (a range of 20–68), and 17 interviewees (47%) were male.

**Table 2.** Respondents’ Characteristics.

Respondent	Sex	Age	Occupational field	Participation status
1	Female	67	Human resources	Participant
2	Male	42	Transportation	Nonparticipant
3	Female	60	Education	Nonparticipant
4	Male	30	Legal and social work	Participant
5	Male	52	Photography and architecture	Ex-participant
6	Male	60	Municipality	Nonparticipant
7	Female	37	Home maintenance	Ex-participant
8	Male	26	Warehouse maintenance	Participant
9	Female	59	Retail	Participant
10	Male	33	Entrepreneur	Participant
11	Male	20	Student	Participant
12	Female	53	Law	Participant
13	Male	30	Student	Nonparticipant
14	Male	66	Mechanical engineering	Participant
15	Male	55	Home maintenance	Nonparticipant
16	Female	29	Publisher	Nonparticipant
17	Female	50	Entrepreneurship	Nonparticipant
18	Female	68	Retired	Participant
19	Male	54	Management	Participant
20	Female	27	Energy policy	Nonparticipant
21	Female	39	Health care	Participant
22	Female	50	Health care	Participant
23	Female	27	Human resources	Nonparticipant
24	Female	46	Education	Participant
25	Male	45	Municipality	Participant
26	Female	65	Farming	Nonparticipant
27	Female	43	Librarian	Ex-participant
28	Female	51	Social work	Ex-participant
29	Female	37	Management	Participant
30	Male	33	Engineering	Nonparticipant
31	Male	60	Human resources	Nonparticipant
32	Female	33	Home maintenance	Nonparticipant
33	Male	45	Management	Participant
34	Female	63	Retired	Participant
35	Male	34	Business development	Nonparticipant
36	Male	49	Entrepreneurship	Nonparticipant

We recruited interviewees partly from the Lifelines biobank. Lifelines is a multidisciplinary population-based prospective cohort study focusing on the health and health-related behaviors of persons who have been living in the Northern Netherlands since 2006. The study has a unique three-generational design and employs a broad range of investigative procedures for assessing the biomedical, sociodemographic, behavioral, physical, and psychological factors that collectively contribute to the health of the general population and to incidences of disease, with a special focus on multimorbidity and complex genetics (Scholtens et al., 2015). The recruitment of new participants for the Lifelines project stopped in 2013. To ensure their privacy, participants and ex-participants were recruited by the Lifelines organization. Ex-participants who had ended their Lifelines’s participation in the last 2 months prior to the commencement of our study in August 2016 were invited by phone to participate in our study. The invitation was communicated by Lifelines in the last stage of the withdrawal procedure of Lifelines, but the invitation protocol was written by the researchers of this study (R.B. and J.A.). Moreover, nonparticipating partners of the Lifelines participants were invited by the organization. Ex-participants were the least willing to participate in our study. Although we invited ninety such individuals, only four consented to participate (4.44%). This response rate was significantly lower than those of participants and nonparticipants (22.2% and 24.9%, respectively). The reasons given by invitees for not participating in our study were “no interest,” “not available,” and “not willing to invest more time in Lifelines or its studies.” Although our sample of ex-participants is too small to allow for generalizations, we nevertheless obtained the in-depth information that we sought. All nonparticipants were members of the public whom we recruited through personal face-to-face invitations issued at the entrance of a central public library and through personal or general written invitations that followed the same invitation protocol issued via various social networks as well as on online and offline platforms. Our recruitment and sampling was in accordance with the approval of the Medical Ethics Review Board.

### Data Analysis

We analyzed each individual phrase of all 36 interview transcripts in the context of the overall interview content, and, where appropriate, we generated or assigned content-related codes. Codes could be applied multiple times in a single transcript, and phrases could consist of multiple codes. Codes with related content were grouped together, and these groups were subsequently categorized into themes. We developed an initial coding protocol after conducting a close reading of three transcripts. This coding protocol was evaluated by two or three researchers working on each transcript through an iterative process of cross-checking the individual transcript analyses. After consensus had been reached about the content of codes, groups, and themes, the resulting coding protocol was finalized and used for the remaining transcripts. There was overall agreement on the coding. When different codes were used, consensus was reached through discussion. Five researchers were involved in coding the transcripts. Six random transcripts were coded by at least three researchers. Subsequently, one researcher coded all of the transcripts, whereas the other researchers coded a limited number of transcripts. In line with the protocol, the results of the interviews were categorized into four themes for ex-participants (EP), participants (P), and nonparticipants (NP). One of these themes, “Attitude toward centralized data repository and linkage in the context of biobanking,” was influenced by our topic guide, given the focus of discussions during the interviews prior to the analysis. The prominence of the other themes did not emerge directly from our topic guide but was revealed by the data. The validity of this process of identifying themes is confirmed by the adaptive theory on qualitative research, which allows for such theoretical influence within research (Layder, 1998). Transcripts were primarily analyzed using a qualitative data analysis software package, Atlas TI version 7, to enable the retracing and evaluation of quotes along with their codes, groups, and themes (Friese, 2013).

## Results

We identified multiple conditions that relate to experiencing the trust required to provide personal data in the context of biobanking and centralized large-scale data repository. However, interviewees found it difficult to explain the reasons for this trust. For example, one interviewee (NP17) provided the following explanation: “I can’t express it in words; it’s a feeling.” Our analysis of the interview transcripts revealed four major themes that featured in the formation of trust and in decisions made by individuals about sharing their data. The first theme that emerged from the data of unique quotes was data repository aimed at benefiting the public. A second theme was organizational aspects of the biobank as a data collector, focusing on how the biobank is organized to provide a context in which data are used safely. The third theme was characteristics of the collected data, which concerned the type of data requested and its relevance for research. The last theme was control over personal data maintained through informed consent. This theme related to the influence of interviewees’ perceptions of informed consent in line with their intention of maintaining control over the personal data that they provided.

### Data Collection Aimed at Benefiting the Public

The interviewees characterized the context of medical scientific research as a quest aimed at improving public health. Although ex-participants as well as several nonparticipants expressed a lack of trust in public goods, and especially in medical scientific research, participants and the majority of nonparticipants perceived the context of medical scientific research to be generally trustworthy and affirmed their trust in Lifelines as a public good. For example, Lifelines was widely perceived as a charitable organization, as observed by one nonparticipant (NP13): “If I understand the question correctly, for me it is actually almost [like] a charity, in which I would participate. [But] instead of money, I give my time.” Participants placed considerable value on the objective of benefiting the public. Several interviewees, mostly participants, stated that they would be unwilling to provide their personal data if the research was primarily aimed at providing financial gains for just a small group of people, such as the managers or shareholders of Lifelines. One participant (P4) stated, “Well, I think it’s different. If it is for a [for-] profit organization then I think . . . look, that’s not why I participated in it.” Another participant (P14) provided the following explanation:

I see someone who wants to sell a washing machine differently from someone who wants to collect data about a certain disease in a certain family. Because the first one tries to sell a washing machine through data [received] from my laptop. That data is passed on just for personal gain. He wouldn’t otherwise [ask for it]. I don’t have that feeling with Lifelines [as data repository]. . . . Lifelines aims to make someone better, but then your offspring instead of yourself.

By contrast, most nonparticipants and a few participants were less concerned about this objective. They expressed their willingness to share their data in return for personal financial gain if they were interested in the research topic. This was expressed by one of these participants (NP2) as follows:

Yes, if they have a commercial interest, I want them to share their profit as well. Then I’m going to look at it in a different way. If you were to make a profit, I would like you to say so.

### Organizational Aspects of the Biobank as a Data Collector

All four ex-participants stated that they had withdrawn their participation because they were disappointed to some extent in the organizational aspects of the biobank, which contributed to a reduced level of trust. For three of four of the ex-participants, the extensive scale of data repository was a critical factor influencing their distrust because they worried about the use of their personal data in Lifelines and the preservation of their anonymity within such a large-scale project. They perceived centralized large-scale data repository to be valuable for researchers, but this also entailed an increased risk. Two respondents explained,

I believed that it would only be used within the hospital or at the University Medical Center. But it goes further in my opinion. . . . See, when you develop medicine or something like that, you need information about large groups of people. I’ve heard that even secret services and the police are interested in DNA material. There is no other large biobank in existence apart from this one with all of its information. From a commercial perspective, this is of course very interesting. (EP5)

Well I believe your data [at Lifelines] are no longer anonymous. I just don’t believe that [data are anonymous] anymore. That doesn’t concern me a lot. Only you don’t know which way things are going. . . . You never know where your [medical] data is going to end up and how it will be used or valued. (EP27)

All participants and most nonparticipants believed, however, that the larger renowned institutes were more competent than smaller institutes because they perceived them to be “better equipped,” “more solidly financed,” and “more experienced in handling their participants’ information.” They did not worry about privacy threats within Lifelines because of the substantial number of participants who would be affected if data were to be leaked or misused. As one participant (P10) observed, “The bigger it gets, the more anonymous [it becomes].” They assumed that data were secure. As one nonparticipant (NP16) explained,

No, well I just assume that the information will be anonymous and wouldn’t become public. . . . That is something you may expect not to happen. You need to take care of security breaches or data leakages when designing such large-scale research.

Moreover, the ex-participants had expected more effective communication regarding privacy, aims, and feedback on their results. Their perception of the lack of proactive communication of the organization and its representatives was the final factor prompting their distrust. One ex-participant (EP7) described,

You have to hand in blood and urine, but you never get an answer [feedback on results]. Yes, if it’s really bad, they will answer, but by then it is too late. Otherwise they won’t give me an answer, for example “That’s good and that’s for the lungs and the heart.” They don’t answer that. . . . You don’t get an answer on whether the heart is still good or not.

All of the participants and nonparticipants indicated that their trust was also based on the anticipated quality and motives of the employees or other representatives of the organization. They mentioned that representatives’ competencies, measured, for example, by the expected university degree qualification, influenced levels of trust. Moreover, “professionalism” signaled trustworthiness. Such perceptions of “professionalism” were based on the formal and consistent behavior of the biobank representatives (e.g., being friendly and calm). This was explained by one respondent (P8) as follows: “It is true that they always take a look at you; they are interested, so to speak. . . . I think that it is very important to show an interest in the people who are visiting.”

None of the ex-participants, participants, or nonparticipants had a comprehensive understanding of how security measures and purpose limitations for data repositories are applied in medical scientific research in general, and specifically by Lifelines. This was a factor influencing the development of distrust or trust among interviewees. In light of their negative experiences that reinforced their personal beliefs, the ex-participants (EP7 and EP8) associated Lifelines with poor medical research and care and with (pharmaceutical) business (EP5 and EP27). As one of them (EP7) explained, this perception was based on information acquired indirectly:

I heard rumours in the newspapers and so forth that some general practitioners had stated that one should not participate in it. Moreover, I heard via an acquaintance who works in IT and was involved partly [in Lifelines] that it works a bit like that [business].

Neither of the above assumptions is backed by objective evidence. Three of the ex-participants (EP5, EP27, and EP28) and some of the nonparticipants were convinced that Lifelines was unable to guarantee the anonymity of the data, which could result in potential misuse of data by the government or by health insurance companies. As one nonparticipant (NP23) observed,

I wonder what a government does with this kind of information. They are not doctors or hospitals, you know. . . . So what do you want to do with them? I can also imagine that they would like to share this with health insurers or people like that. Then I think, sorry, but if that means that in 10 years’ time, for example, I will need enormously expensive medicines, then my health insurer doesn’t need to know. So it depends a bit on who and for what purposes.

Lifelines provided participants with information about security measures and strictly regulated methods that were deployed at the onset and during their participation via information leaflets, informed consent forms, and newsletters. However, none of the participants could explain in detail how their donated material was being used, stored, and shared. One participant (P10) stated,

What you see and the information you get, that will be sufficient. Yes, it sounds very naive, but you can’t really do it. . . . Going there on one visit, you’re not going to ask: “Show me what you’re doing.” No, you don’t do that. You just assume that this is good.

As one participant (P4) clarified, the biobank was perceived as trustworthy because of its link with the University of Groningen and with the University Medical Center Groningen:

I believe that the University Medical Center is the mother of the organisation. That is one of the most important organisations in the medical sciences, here in the North. . . . That scientific element of the research and the organisation is at the top of my list of factors that play a role. Well, if it wasn’t scientific, not linked to the university, then I would have to think about it again perhaps.

### Characteristics of Collected Data

Interviewees mentioned that the sensitivity of the data that they provided, and hence the level of trust required, was influenced by the range of collected data. Interviewees suggested that the aim of the research should match the data requested. Two ex-participants stated that the broad range of data collected by Lifelines was an important concern for them influencing their withdrawal as participants. In their view, if, in addition to medical details, the data encompassed supplementary information on aspects of their personal lives, such as their financial information, then such data would be more sensitive and misuse of the data could have a significant impact on their lives. According to one ex-participant, it could be explained as follows (EP27): “before that [negative experiences during visits], I had seen a documentary about privacy. So that may have been one of the factors because you don’t know what the consequences of [sharing] your information are.” All of the ex-participants and most of the nonparticipants had concerns about the possible abuse of their data in the future and were wary about providing data to the biobank, especially via data linkages. Some of them felt that a future trend of organizations controlling people’s lives was likely. For example, one nonparticipant (NP15) stated, “But if you have a lot of data, then you can manipulate people. . . . In the end, you can use every piece of information about someone to control him or her.” The distrust of these interviewees mainly stemmed from centralized large-scale data repository outside the scope of scientific research, such as data extraction from social media or web browsers, and especially search engines. However, they also mentioned other elements of distrust relating to such data repository in the context of medical scientific research. Thus, a participant (P1) made the following observation: “Well a very good example is that this knowledge will enable you to potentially exclude people from insurance. . . . That is something that frightens me.”

These concerns were also shared by other interviewees to a lesser extent. One nonparticipant (NP16) described the following concern: “If they acquire data on other aspects of our lives, then they truly become ‘Big Brother’ . . . they can really dig into your life.” More specifically, these interviewees were concerned about the possibility of the Lifelines database being linked to data held by institutes that did not focus on health care, for example, Statistics Netherlands or the Dutch Tax authority. This concern is expressed in the following quote:

For example, my tax information does not seem to me to be relevant for Lifelines. That is my intuitive feeling. If someone could explain to me why this information is relevant, that could change my opinion. However, my primary response is that it is not necessary to provide such information. (NP30)

The interviewees who had concerns about centralized large-scale data repository expressed doubts as to whether their personal data would be used in a way that they perceived as nonthreatening in the future, given Lifelines’s linkages with such institutions.

Yes, they can dig into all your facets of life. Of course, medical information is already quite personal. . . . The most personal thing you can know about someone. But if they also want to see your pay check and so on, it might well be a matter of concern. (NP16)

Several nonparticipants and some participants, however, perceived opportunities in addition to risks relating to participation in research and the linking of personal data. Their interest in and support of medical scientific research were decisive factors influencing their consent to participate in this centralized large-scale data repository. They stated that they would supply a broad range of requested information providing that certain conditions were met, such as adherence to their informed consent and its importance in relation to a relevant research context. One participant (P17) stated that “if they could make it clear to me why that is important, I would consider it. I wouldn’t just say beforehand ‘oh yes, just take it.’” Another participant (P9), who had reservations, observed, “Well, if it is about your well-being and your health, then I think keep [the data request] to that theme.” These participants mentioned having a gut feeling that something could be wrong with data sharing in general or that their privacy could be threatened after discussing their data contributed to the biobank. These apprehensions mainly stemmed from their conception of a future entailing an increasing risk of data being misused by organizations to influence behavior. One participant (P1) provided the following explanation:

Many people do these things and what they will do with that huge mountain [of data]. . . . I fear no one knows anymore what can be done or what cannot be done with it. There are no inputs into this matter, so to speak. So . . . abuse is also obvious or conceivable.

Despite these apprehensions, however, the interviewees felt that the provision and linkage of their data to the biobank was inevitable, given the high degree of relevance of their medical information. A participant (P4) stated,

It is a bit scary. Nowadays they want to know everything about you, and even the government knows where you are, where you drive, where you check in with a public transport card. But yes, that is also the time we are living in. So I think we should go along with that.

None of the other participants had any reservations about the increasing provision and linking of their personal data to the biobank via other organizations because they had nothing to hide within the specific context of human research in medical sciences. The objective of health care justified their full contribution to the biobank and to medical scientific research. Two participants explicitly stated that if Lifelines considered financial information necessary for research purposes, then they would provide it without stipulating any conditions:

How far can they go with that [data collection]? Extremely far for me. . . . if that requires me to reveal sensitive things, like what diseases I have had, how my sex life is or how much I drink, no problem. Even if they put it on the Internet, if I [help to] make someone better with it [the information], then they should do that. (P10)

Another participant (P11) provided the following explanation:

You give away data, and for the rest, you are not 100% sure if anyone will ever leak something . . . or that . . . it will be used against you ever. . . . But I don’t attach a great deal of importance to that if I compare that with what kind of benefits there can be, so to speak.

### Control Over Personal Data Maintained Through Informed Consent

The interviewees’ subjective perceptions of informed consent given to the biobank played an important role in their decisions to withdraw their participation or contribute data. All of the ex-participants indicated that their reason for withdrawing was to take back control over their data because they felt that they had been misinformed or given insufficient information. One of them (EP5) stated,

Well, I couldn’t find anything on the Internet, for example, from the Lifelines organization on what exactly they do with all the information. That was nowhere to be found. Then I thought, what’s secret? . . . I believe that journalists have been looking for that [information] too. I couldn’t find anything about it. . . . I think they should disclose that much more. What exactly do they do with it? What exactly is their goal?

Thus, they lost trust not only in the biobank but also in data sharing in general or in health care. Their trust diminished after they were misinformed about data management or the nonprofit policy, or when they developed a distrust of data sharing in general. It also diminished after they gained access to new information or exposure to media debates about research and data security. Although some interviewees from the group of participants had given their consent, they were still somewhat hesitant to provide their personal data because of a perception of loss of control: “You don’t have any control. . . . My problem concerns what they do with it. You have to have that confidence in them, which I find a bit difficult.” (P3)

One third of the interviewees, mainly participants, mentioned that providing personal data to the biobank would not be an issue because informed consent had been or would be given. They felt that they had adequate control over their donated personal data and could “always” retract their data if necessary:

If I must think about everything, that’s simply not me. “Oh, what is he going to do with my data?” . . . I don’t care. I just don’t say things that I don’t want to tell them. (P22)

Nonparticipants appeared to be more cautious and valued the personal data they would provide as sensitive. For example, one nonparticipant (NP3) explained,

Look, if I am part of a group, and I have to make a choice. . . . I have no control. . . . What concerns me is what they do with it. You must give them that trust, but I find that a bit difficult. In my opinion, it is the same with politics. Municipal politics, I find that easier. Otherwise it’s [politics] too broad.

Although views on the value of the contributed personal data differed, the procedure of informed consent was widely valued as an important symbolic act. Although four interviewees (two participants and two nonparticipants) acknowledged that they themselves could not verify how data were managed at Lifelines, they still preferred to retain some form of control over what happened with and to their data.

## Discussion

Our aim in this study was to understand why and how people are willing to entrust their data to a facility engaged in centralized large-scale data repository for human research and what conditions would contribute to a reduction in their trust. Accordingly, we focused on possible differences in trust levels and in perceptions relating to data sharing among ex-participants, participants, and nonparticipants in a Dutch population-based biobank. Our results showed that evaluations of the biobank were related to its aim of being a public good and its grounding in medical science. Questions about its aims and nature as a public good and concerns about privacy seemed to be key factors influencing the withdrawal of participation and the building of trust. We found that the following four factors influenced trust building relating to centralized large-scale data repository for human research: data repository aimed at benefiting the public, the organizational aspects of the biobank as a data collector, characteristics of the collected data, and maintaining control over personal data through informed consent.

Our results suggest that the medical scientific context is a key factor that influences the development of trust in biobanks and researchers in two ways. First, medical scientific research was generally perceived as trustworthy by participants and nonparticipants because it was considered beneficial for society, with several respondents perceiving a population-based biobank as a charitable organization. In other words, the underlying purpose of the scientific medical field contributes to the trustworthiness of research organizations. Previous studies have shown that diminution of trust in providing data can be related to commercialism (Critchley et al., 2015; Nicol et al., 2016; Steinsbekk, Myskja, & Solberg, 2013). Our results showed that this was indeed one of the main concerns about participation, especially relating to its withdrawal.

Second, the context of medical sciences was perceived as trustworthy, entailing qualified organizations and representatives. This perception reveals the importance of standardizing and harmonizing research systems based on clear principles, rules, and codes of conduct. It is nevertheless important to acknowledge that the contexts of medical science in other countries may be quite different and may not elicit similar degrees of trust (Sheikh & Hoeyer, 2018). Moreover, this factor has a bearing on the interpretation of the value placed by interviewees on informed consent. Our findings indicate that despite lack of recall of its content, informed consent was perceived by ex-participants, participants, and nonparticipants as a symbolic act to which a high moral value was assigned (Allen & Mcnamara, 2011).

The level of the interviewees’ trust in centralized large-scale data repository or biobanking was also influenced by the organizational aspects of the biobank as the data collector. The large scale of data repository and the sound reputation or trustworthiness of the responsible organization motivated participants and some nonparticipants to provide personal data despite their lack of knowledge regarding current security measures. Although providing data for Lifelines may entail risks concerning individuals’ privacy and autonomy, most participants and nonparticipants experienced a sense of safety relating to the number of other participants in the biobank. By contrast, the large scale of data repository elicited a higher degree of distrust among ex-participants and several other nonparticipants. Previous findings indicate that the perception of strength and safety in numbers accounts for one of the reduced risks and increased positive outcomes (Park & Hinsz, 2006). This finding of difference in trust is in line with that of previous studies, suggesting that differences between participants and nonparticipants may be attributed to predisposed differences in terms of their concerns or generalizations of trust. Such differences have a bearing on information collection procedures as well as on the acceptance of new methods for collecting data (Nobile et al., 2016).

The trustworthiness of the biobank as an organization was evaluated by the interviewees partly on the basis of their perceptions of the motives, behaviors, and competence demonstrated by employees or other representatives of the biobank. This finding endorses previous findings that interpersonal relationships with health care representatives, such as general practitioners or biobank employees, matter in people’s decisions to participate in research (Johnsson, 2013). The behaviors and communication styles of employees or other representatives of biobanks constitute the missing link, accounting for diminishing trust, as demonstrated by the ex-participants. This finding may indicate that trust in centralized large-scale data repository is linked to perceptions of the trustworthiness of familiar entities (O’Neill, 2018), such that the aims and competencies of representatives are equated with the claims of the organization. Employees and representatives seem to be the gatekeepers of the bridge that stretches from trust, at one end of the spectrum, to distrust at the other. A lack of professionalism on their part is the final factor contributing to diminution of trust and the decision of individuals to withdraw their participation.

Ex-participants, nonparticipants, and even some participants had reservations about the process of combining information and data acquired from different contexts. The finding of the importance of the characteristics of the data, that is, their sensitivity and relevance to the research topic, highlights the significance of the context when collecting data or linking data sets. This context is highly pertinent when considering the willingness of individuals to provide personal data. This challenges trends of centralizing data repositories in human research by internal and external linkage to other data sets, in particular to data sets about nonmedical contexts. Trust in a particular context and within organizations is contingent on the aims and norms that are valid in one context and is, therefore, of limited effectiveness in reducing complexity when attempting to optimize centralized large-scale data repositories. These findings are in line with scholastic views on privacy that consider context as a key factor underlying concern (Barocas & Nissenbaum, 2014). Moreover, they support the bioethics guidelines formulated in the United Kingdom that state that the sensitivity of data is contingent on the context in which they are used and related to other data (Nuffield Council on Bioethics, 2015).

The findings of this study confirmed that “blind trust” on the part of participants in human research organizations was induced by the “halo effects” of the responsible organization and context (Hall et al., 2001). All (non)participants generalized their trust or distrust in the biobank and its context relating to data linkages to simplify the decision of contributing personal data. The level of risk associated, for example, with data optimization through linkages of data in a new setting can be estimated based on the experiences of interviewees combined with the perceived norms and values of the research organization or context. The role of trust therefore assumes prominence in decisions on whether or not to participate in research as a strategy for reducing complexity (Luhmann, 1979), particularly when the nature of data repository becomes increasingly complex.

In general, the perceptions of ex-participants, participants, and nonparticipants on data sharing varied, with most participants being generally positive about centralized large-scale data repositories, focusing on opportunities that they offer rather than on threats. In contrast, nonparticipants and ex-participants had more concerns relating to these collections and offered reasons for these concerns. The findings of studies conducted among potential participants—4,659 and 15,650—in the United States and Europe, respectively, revealed similar attitudes (Gaskell et al., 2013; Kaufman et al., 2009). Although 90% of potential participants within a large cohort study in the United States expressed concerns about privacy, 60% of them stated that they would still participate in the study. The findings of the European study revealed that privacy concerns were less relevant to the intention to participate because of trust in key actors, for example, governments and universities. Therefore, the extension of existing trust seems to be an automatic process, whereas establishing trust from scratch could entail a cognitive rational process. This finding indicates that trust and distrust are distinct concepts in terms of structure. However, they are equal in terms of their functions and therefore feature in decisions about risk taking (Luhmann, 1979). This finding is in line with those of previous studies suggesting that participants and nonparticipants differ in intrapersonal characteristics, such as generalized trust and distrust, which have relevance for information collection procedures as well for accepting new data repository methods, such as big data (Nobile et al., 2016).

In addition to highlighting relevant factors of (non)participation and trust in large-scale medical studies, our findings raise some ethical questions about current responsibilities and mechanisms for protecting individuals’ integrity. The implementation of principles such as informed consent is highly valued in human research—apart from their being legally required—to protect participants’ integrity (Eyal, 2014; Tassé & Kirby, 2017), and the implicit assumption underlying these principles is that participants are rational individuals. Our results revealed that when individuals need to decide whether or not to provide information to a biobank, feelings of trust in general, the research context, and the reputation of the organization seem to be more relevant than knowledge about potential threats to their personal integrity. These factors may be even more relevant to decisions relating to the acceptance of centralized data repository and data linkages.

The above discussion foregrounds the ethical issue of self-determination through one instance of detailed informed consent conceived as the basis of the consent and information process, particularly in relation to biobanks, which are public entities. When biobanks are perceived as charitable organizations, the societal context appears to be a prominent factor in the decision to entrust an organization with personal data. However, the current consent and information-gathering procedures mainly focus on risks for individuals that are of a physical nature (Broekstra et al., 2017; D’Abramo, 2015). In light of these issues, our results suggest that current procedures, such as broad informed consent obtained in large cohort studies and biobanks (Steinsbekk, Myskja, & Solberg, 2013), are inadequate for safeguarding participants’ personal integrity and trust. On one hand, the societal context and potential implications of participation, such as reduced privacy, are not completely addressed, and on the other hand, participants are not protected against psychological biases at an individual level.

## Best Practices

The combination of our findings leads to several recommendations for the management of biorepositories and data repositories. First, the findings argue for an adoption of different measures in information and consent procedures, such as models of governance or consent based on solidarity to ensure participants’ autonomy (Mittelstadt & Floridi, 2016; Prainsack & Buyx, 2013). These models can accommodate risks on individual as well as societal levels, given the focus on decreasing potential threats to individual participants versus providing more complete and relevant information for research or other public goods. Second, it is important to design continuous communication strategies for participants and the public while taking into consideration organizational aspects and characteristics of collected data that influence levels of trust, such as scale of organization, quality of employees, or context of collected data. This finding highlights the moral responsibility of the scientific community to engage in public outreach regarding centralized large-scale data repository in scientific human research, especially in the medical sciences.

One strength of our study, which entailed the conduct of in-depth semi-structured interviews, is that it has shed some light on the complex decision-making process for providing personal data to a biobank. To the best of our knowledge, this study is the first to identify and compare factors associated with the trust of ex-participants, participants, and nonparticipants in centralized large-scale data repository for human research. Moreover, through its focus not only on members of the public as nonparticipants in data repository but also on ex-participants, the study has provided a broader perspective on decision making that pertains to the provision of personal data in centralized large-scale data repository.

The study revealed differences in the perceptions, concerns, and elements entailed in trust building associated with the sharing of data among these three groups. This comparison of these three groups is, however, tentative, as the sample size was small, especially for the group of ex-participants. Not surprisingly, most ex-participants who were approached, as well as their partners, were unwilling to participate in our study. The fact that the biobank itself recruited the ex-participants probably decreased their willingness to participate in our study. Importantly, saturation occurred during the interviews held with participants and nonparticipants as well as with ex-participants, suggesting that our study results were not strongly affected by issues of representativeness for any of the three groups.

## Research Agenda

The present study provides some initial evidence relating to the factors that determine individuals’ trust levels and their decisions to withdraw from participation in human research in the field of medical science. Our results point to the relevance of several factors, for example, the importance of capable and trustworthy representatives and the reputation of an organization. Evidently, further research on this topic is needed. For example, it seems worthwhile to focus on the potential role of individual characteristics entailed in generalized trust and the decision to share personal data with biobanks, especially via data linkages. These factors could be investigated more effectively using quantitative methods.

Furthermore, the results of our study suggest that knowledge about risks, safety measures, and the benefits of technologies could raise concerns of privacy and security among some nonparticipants and ex-participants, especially in contexts where trust levels in data repository procedures are low. Proactive communication and information-gathering procedures are therefore critical for avoiding distrust. Two previous studies showed that an inverse relationship exists between perceived risks and perceived benefits, which determined the acceptance of technologies (Siegrist, 2000; Siegrist & Cvetkovich, 2000). Such a relationship was not, however, found for individuals who reported having knowledge about these risks and benefits (Siegrist & Cvetkovich, 2000). The conduct of more such quasi-experimental studies would contribute to an understanding of the effects of such interventions entailing communication and information-gathering procedures. Furthermore, studies should focus on differences among domains of human research to determine which elements are decisive for trust building. Given that we only focused on the medical field, a comparison of different contexts would be pertinent.

## Educational Implications

These findings suggest several courses of action for employees from data repositories in human research, and the researchers themselves. First, trust is enhanced by qualified and trustworthy employees, which may have training and educational implications for biobanks as public goods seeking a large number of participants. Moreover, there is an important role for governments in providing guarantees of the commitments and competencies of institutes and representatives within research systems via training and education (Gilson, 2003). Second, proactive communication is key for preventing diminution of trust, in particular for changes in data collection. Trust and engagement can be enhanced through face-to-face contact (Gaskell et al., 2013). Ex-participants reported that the organization or its representatives did not communicate proactively with them. Prevention of withdrawal of participation could, therefore, be done by being able to provide the right explanation on time, for example, about data security, organizational aspects, or limits of context of data repository.

## Conclusion

In conclusion, the findings of the present study indicate that the decision to provide personal data for medical scientific research and to accept centralized data repository or data linkages is not one that is made easily. It relies heavily on generalized trust, which is built upon factors relating to the context of data repository, but it is also contingent on individuals’ attitudes and values. Recent proposals relating to governance in biobanking (Mittelstadt & Floridi, 2016; Prainsack & Buyx, 2013) and informed consent (Budin-Ljøsne et al., 2017) are heading in the direction of customization and greater transparency rather than focusing on individual participants and their needs. Contexts perceived as trustworthy, dynamic data governance, and informed consent models and ensuring benefits for the public from research may enhance potential participants’ willingness to provide personal data. These concepts foreground reciprocity and interactions between researchers and (non)participants, with the aim of tailoring research within a wider context, thereby contributing to the building of trust in human research. However, challenges lie ahead for achieving the sustainable use of centralized large-scale data repositories in human research.
